# Supplementary material for: Dual-BONCAT reveals distinct subpopulations of anabolically active cells
Source: Appl Environ Microbiol. 2026 Apr 16;92(5):e02391-25. doi: 10.1128/aem.02391-25 (PMC13188899; doi:10.1128/aem.02391-25)

**Supplemental Material for**

Dual-BONCAT Reveals Distinct Subpopulations

of Anabolically Active Cells

Dylan Mankel^a^, Yasheng Maierhaba^a*^, Claire Momjian^a^^,

Federica Calabrese^a^, Solange Duhamel^b^, Jeffrey Marlow^a#^

^a^Department of Biology, Boston University, Boston, MA, USA

^b^Department of Molecular and Cellular Biology, University of Arizona, Tucson, AZ, USA

Fig. S1: Representative fields of view from mono-BONCAT experiments with *E. coli* cultures, evaluating fluorescence signals derived from a range of HPG and Azide-Cy3 (green) concentrations. Cells were also counter-stained with DAPI (blue). All scale bars are 10 µm.


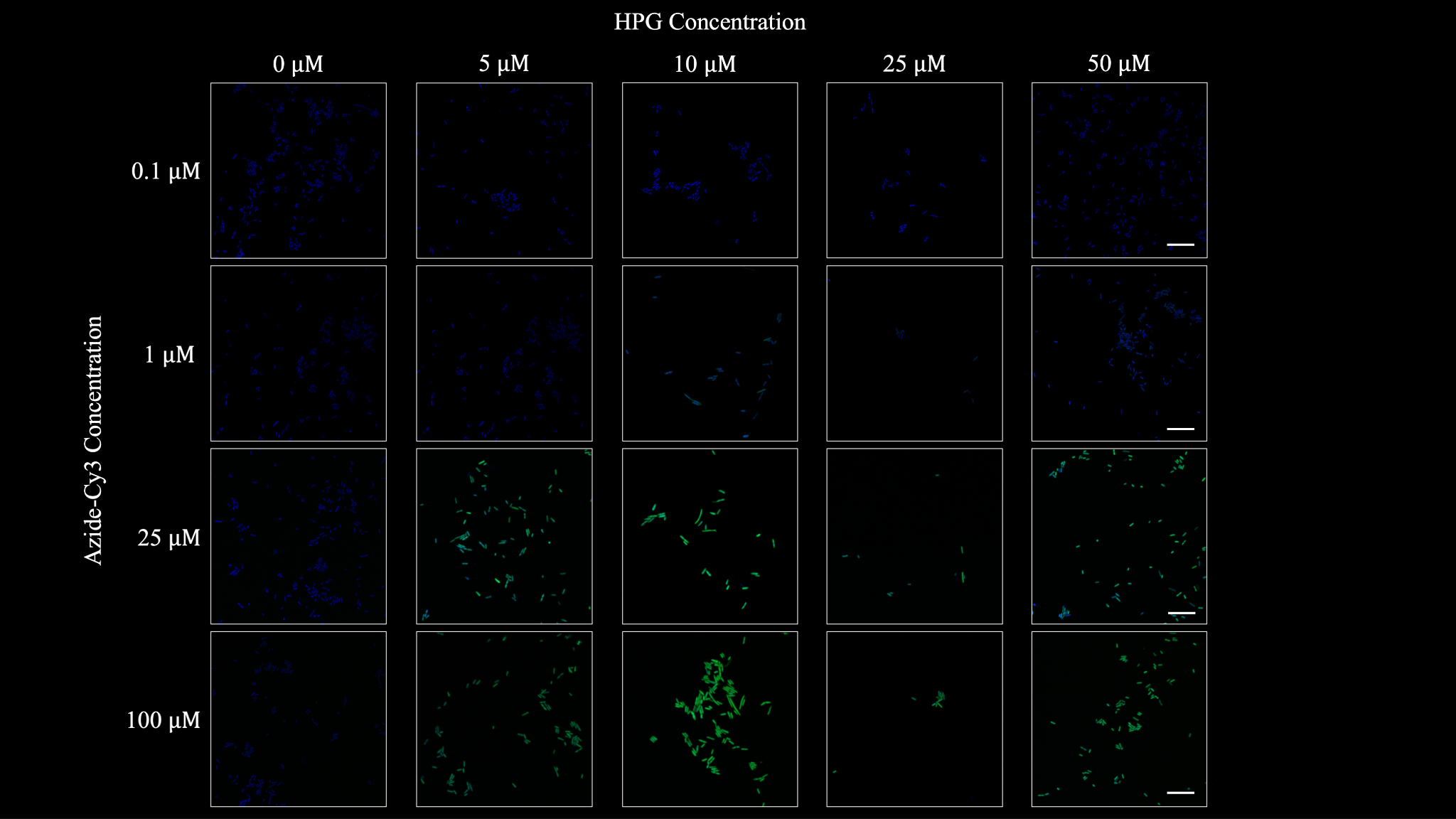


Fig. S2: Representative fields of view from mono-BONCAT experiments with *E. coli* cultures, evaluating fluorescence signals derived from a range of AHA and DBCO-Cy5 (red) concentrations. Cells were also counter-stained with DAPI (blue). All scale bars are 10 µm.


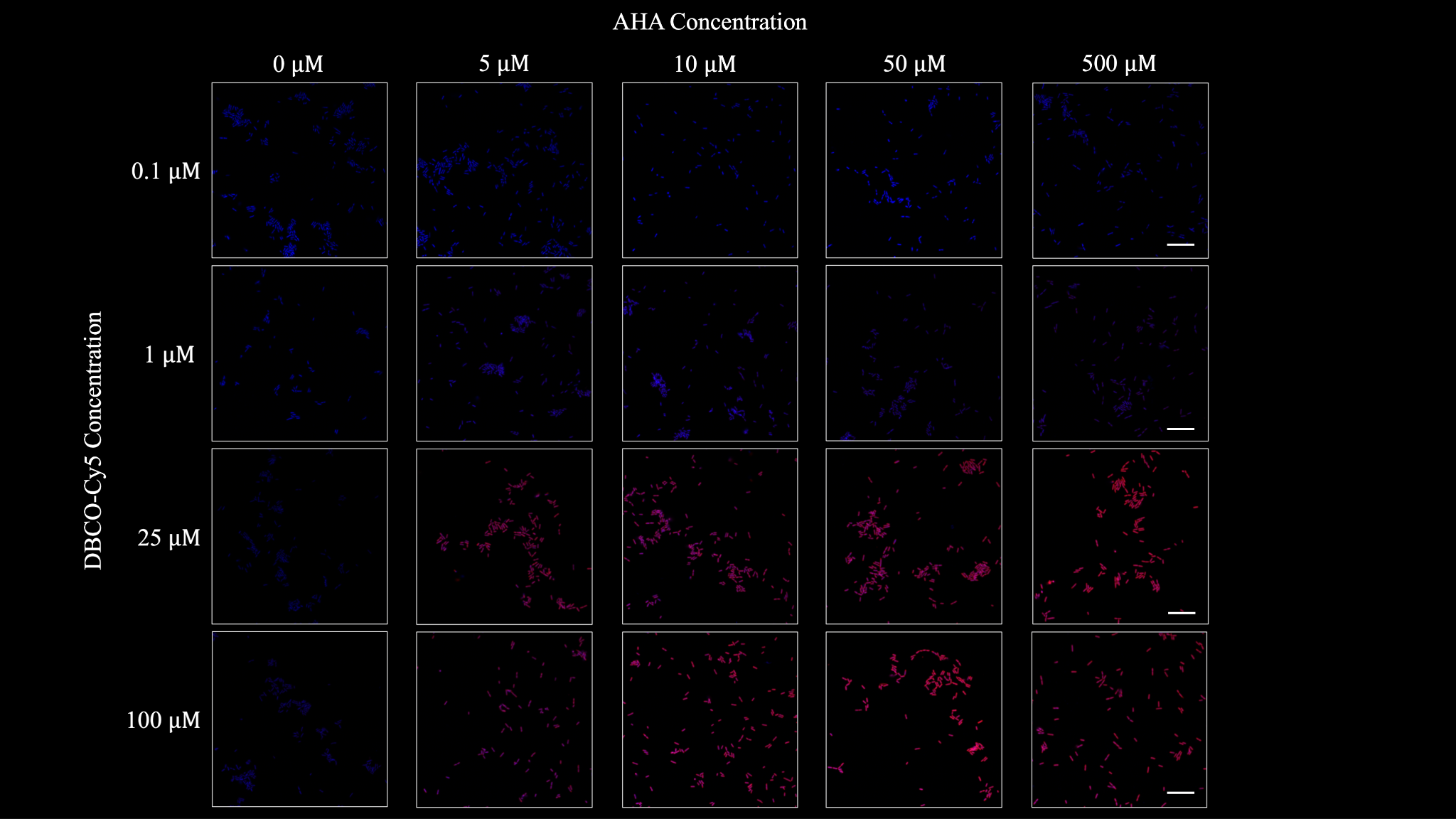


Fig. S3: Boxplots of mean fluorescence intensity values of cells in mono-BONCAT experiments with *E. coli* cultures. Boxes represent the first and third quartiles, while the middle line represents the median. Whiskers extend to the highest and lowest values within 1.5x of the inner quartile range between the first and third quartiles. For each condition, boxes on the left correspond to Cy3 fluorescence and boxes on the right correspond to Cy5 fluorescence. A range of ncAA and dye concentrations were tested to determine parameters for subsequent dual-BONCAT experiments. Imaging parameters were consistent across all different conditions.


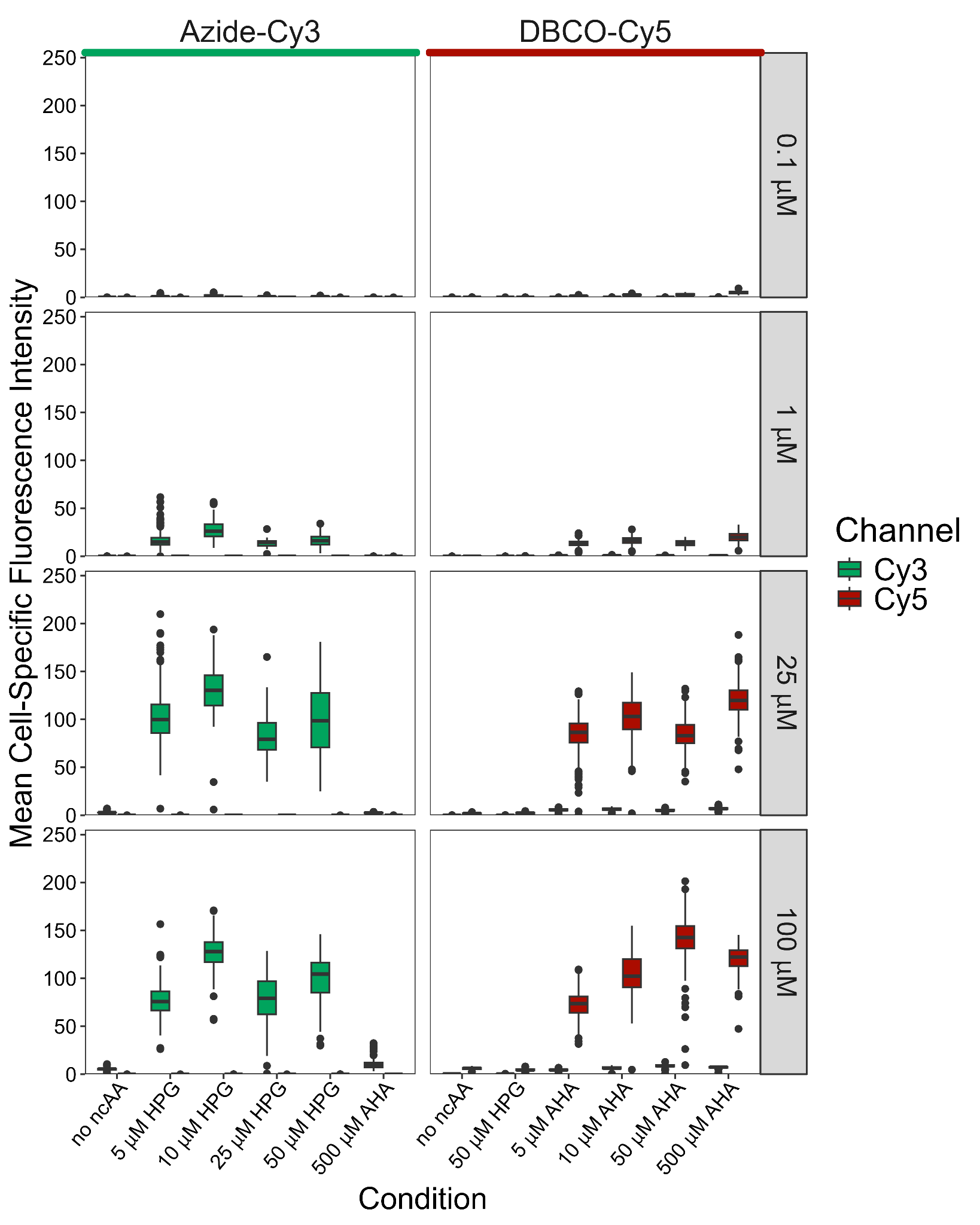


Fig. S4: Representative fields of view from dual-BONCAT experiments with *E. coli* cultures, evaluating the order of ncAA addition and performing the HPG-Azide reaction first followed by the AHA-DBCO. All scale bars indicate 10 µm.


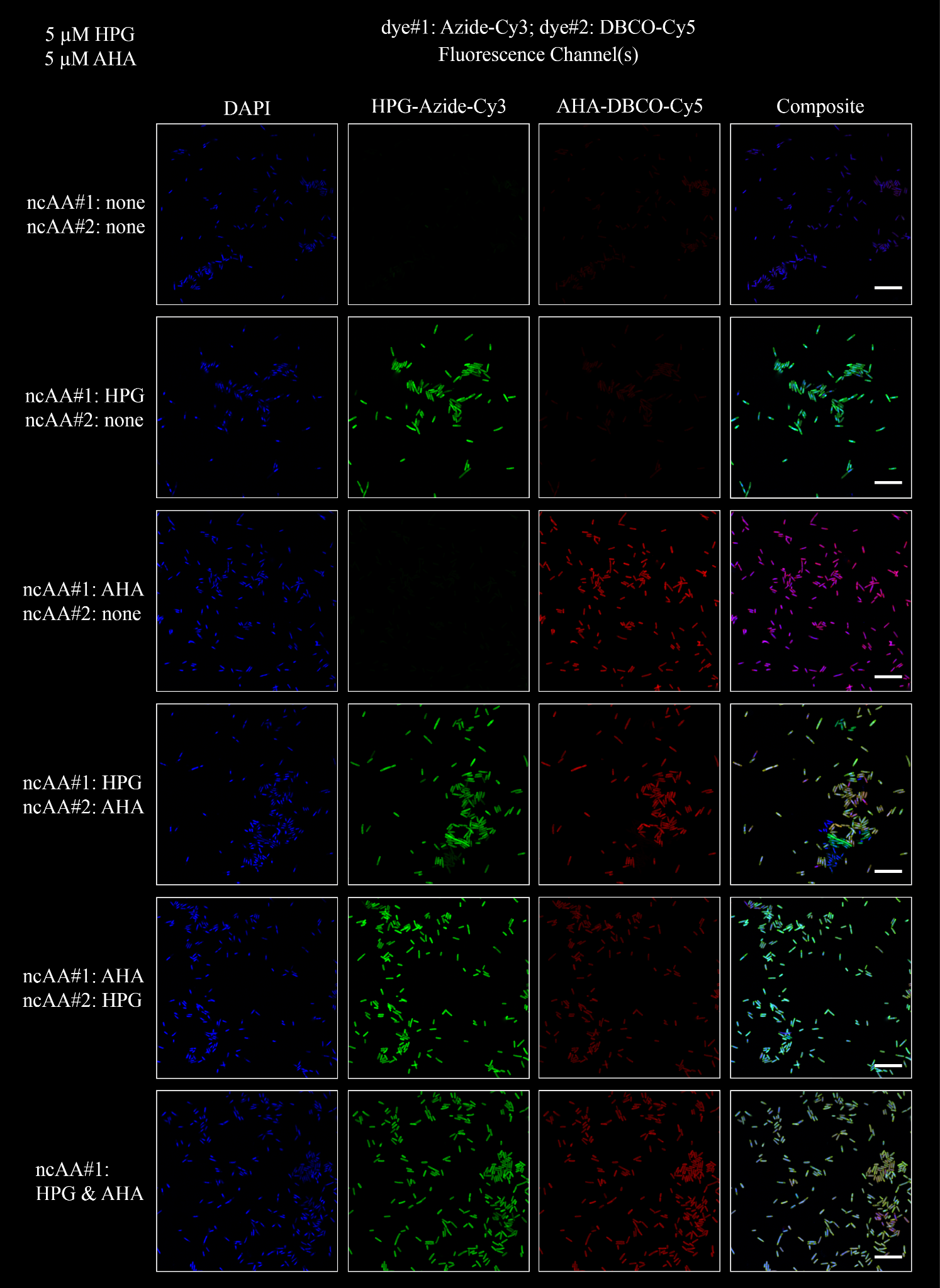


Fig. S5: Representative fields of view from dual-BONCAT experiments with *E. coli* cultures, evaluating the order of ncAA addition and performing the AHA-DBCO reaction first followed by the HPG-Azide reaction. All scale bars indicate 10 µm.


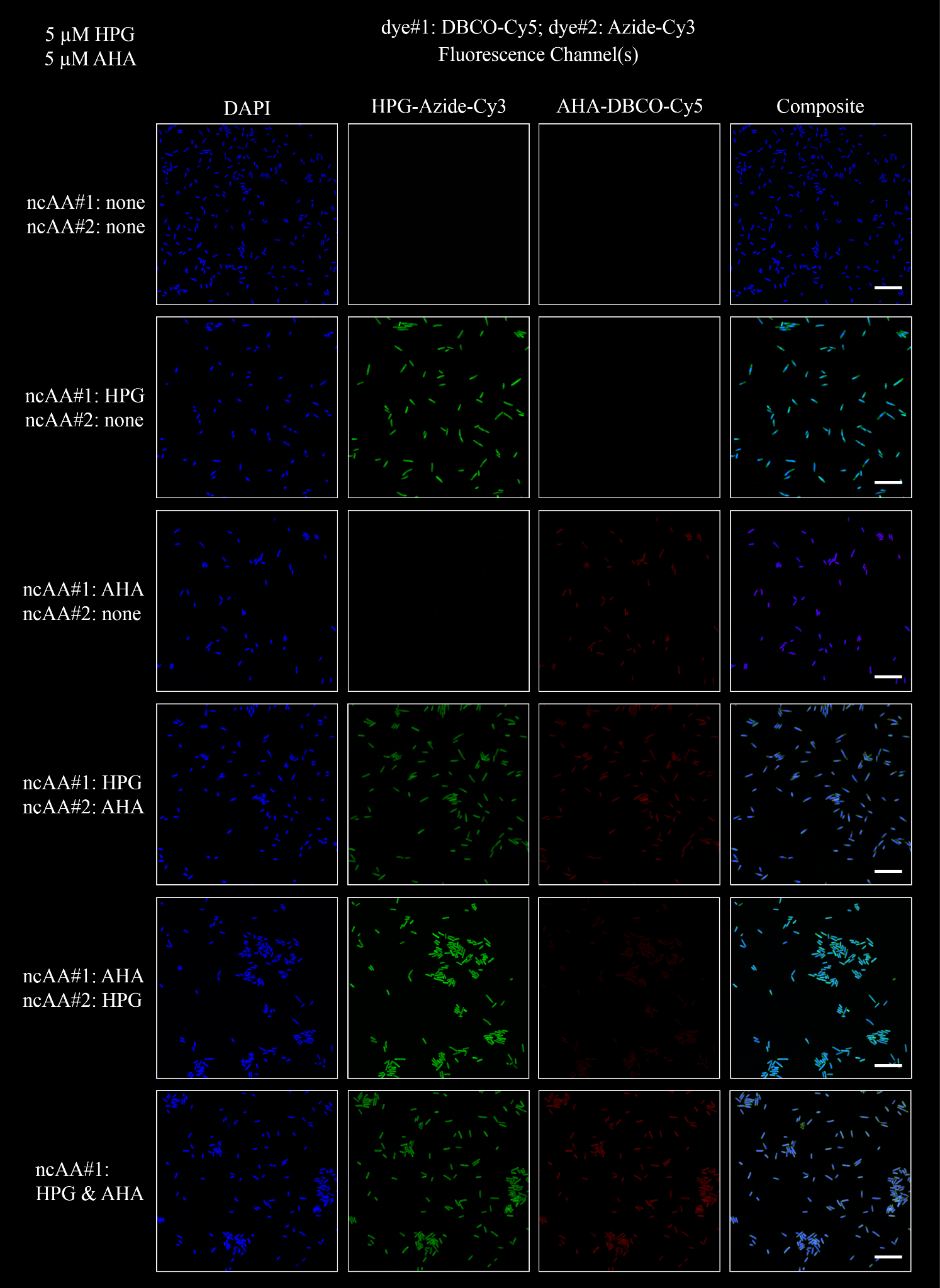


Fig. S6: Fluorescence microscopy field of view of a dual-BONCAT experiment with *E. coli* cells. Both ncAAs were added at the start of the experiment, and, following fixation, the Azide-Cy3 click reaction was performed first. Scale bar indicates 5 µm.


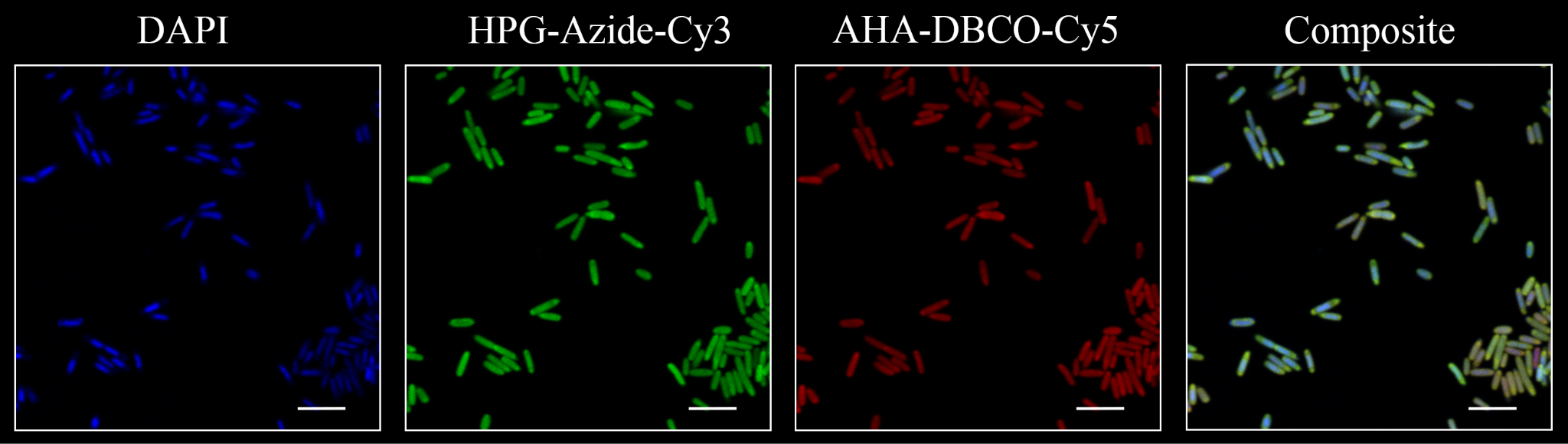


Fig. S7: Ratio of the Cy3 to Cy5 fluorescent intensities for the experiment in which HPG and AHA were added at T_1_ in the field of view chosen for Fig. S6 (left), and the experiment in which HPG was added at T_1_ and AHA at T_2_ in the field of view chosen for Fig. 3 (right). Cells whose mean intensities exceeded the maximum intensities in the “no ncAA” condition were considered labeled. The red-colored bar indicates the cell labeled with a red arrow in Fig. 3; the bar marked with a red asterisk indicates the two cells labeled with red arrows and asterisks in Fig. 3.


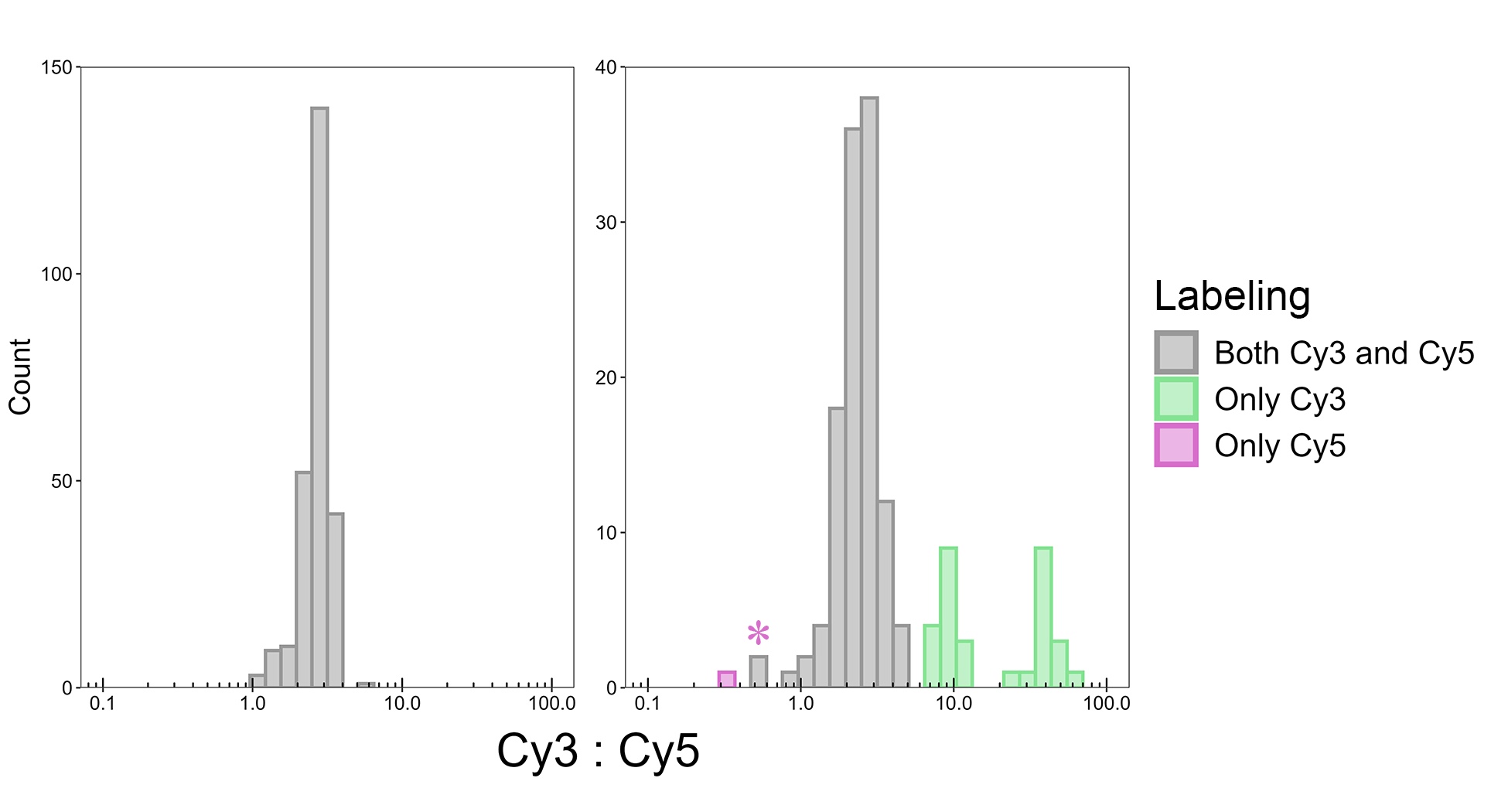


Fig. S8: Representative fields of view from dual-BONCAT experiments with extracted cells from salt marsh sediments, evaluating the order of ncAA addition and performing the HPG-azide reaction first followed by the AHA-DBCO. All scale bars indicate 10 µm.


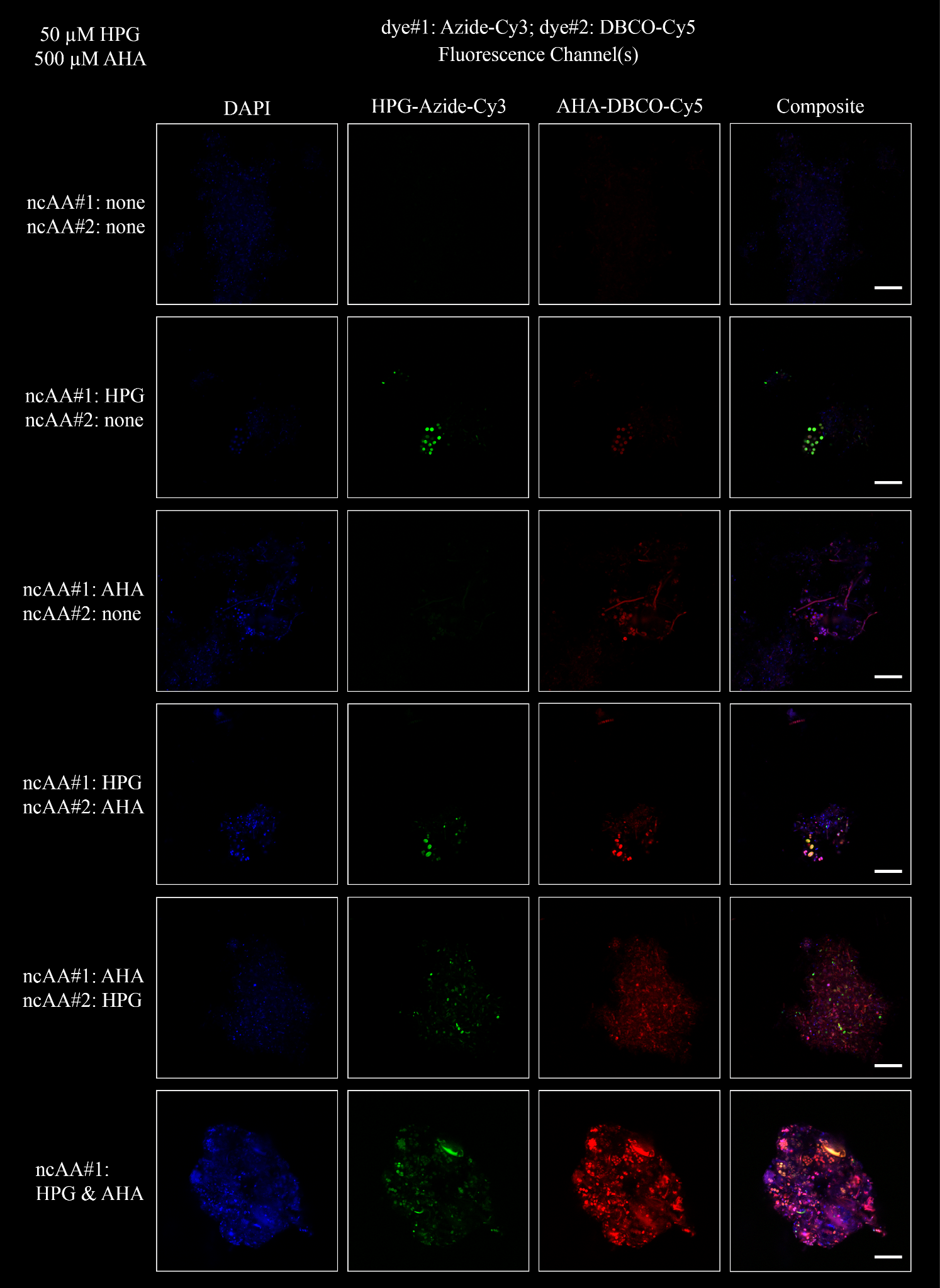


Fig. S9: Representative fields of view from dual-BONCAT experiments with extracted cells from salt marsh sediments, evaluating the order of ncAA addition and performing the DBCO-Cy5 reaction first followed by the HPG-azide. All scale bars indicate 10 µm.

*
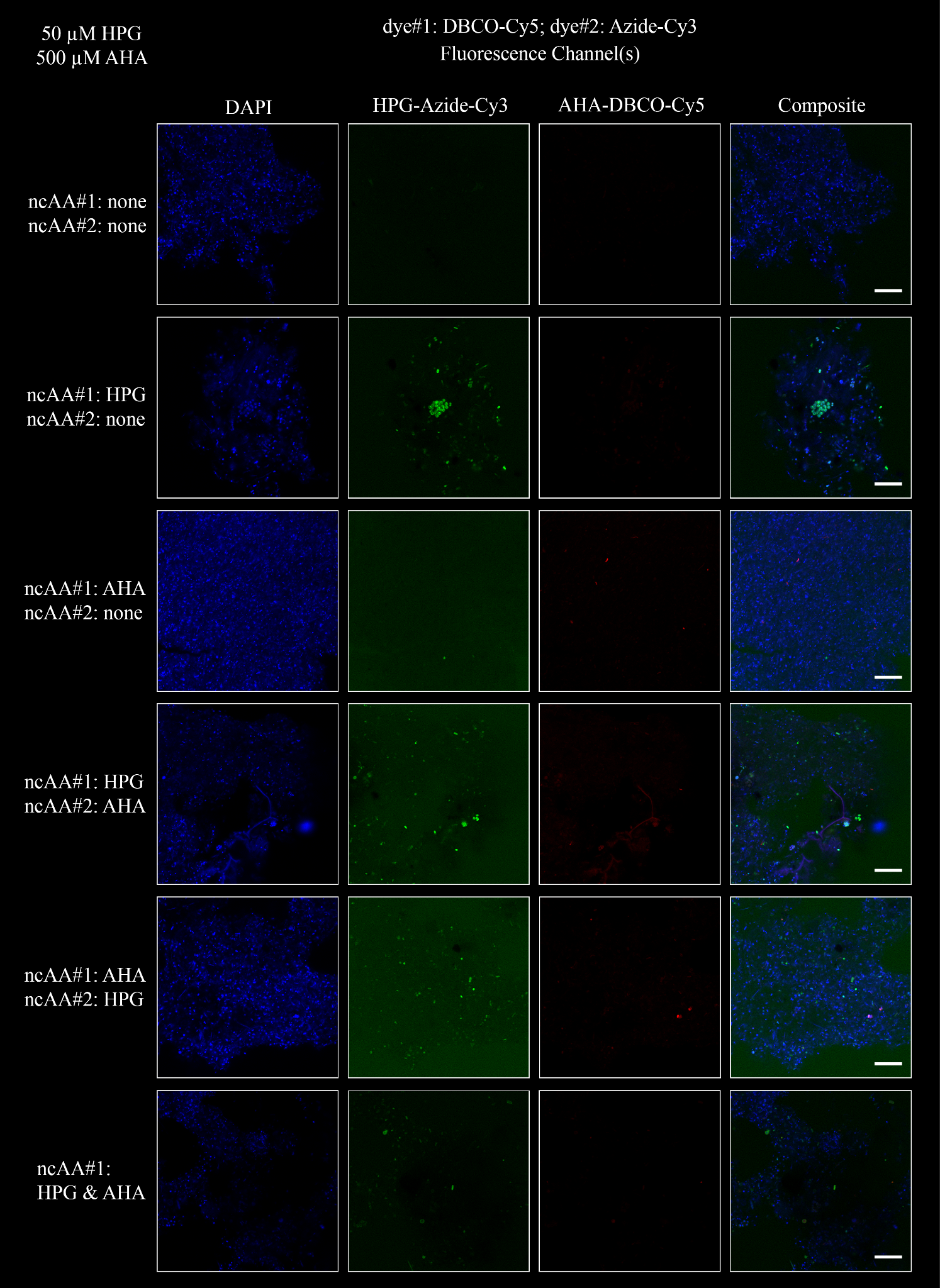
*

Fig. S10: Boxplots of mean fluorescence intensity values of cells in dual-BONCAT experiments with extracted salt marsh sediment cells. Boxes represent the first and third quartiles, while the middle line represents the median. Whiskers extend to the highest and lowest values within 1.5x of the inner quartile range between the first and third quartiles. For each condition, boxes on the left correspond to Cy3 fluorescence and boxes on the right correspond to Cy5 fluorescence. Tests varying the sequence of ncAA addition (see column labels along x-axis) were performed, with the Azide-Cy3 click reaction conducted first (left panel) and with the DBCO-Cy5 click reaction conducted first (middle panel). Data from the 5 µM HPG & 5 µM AHA condition.


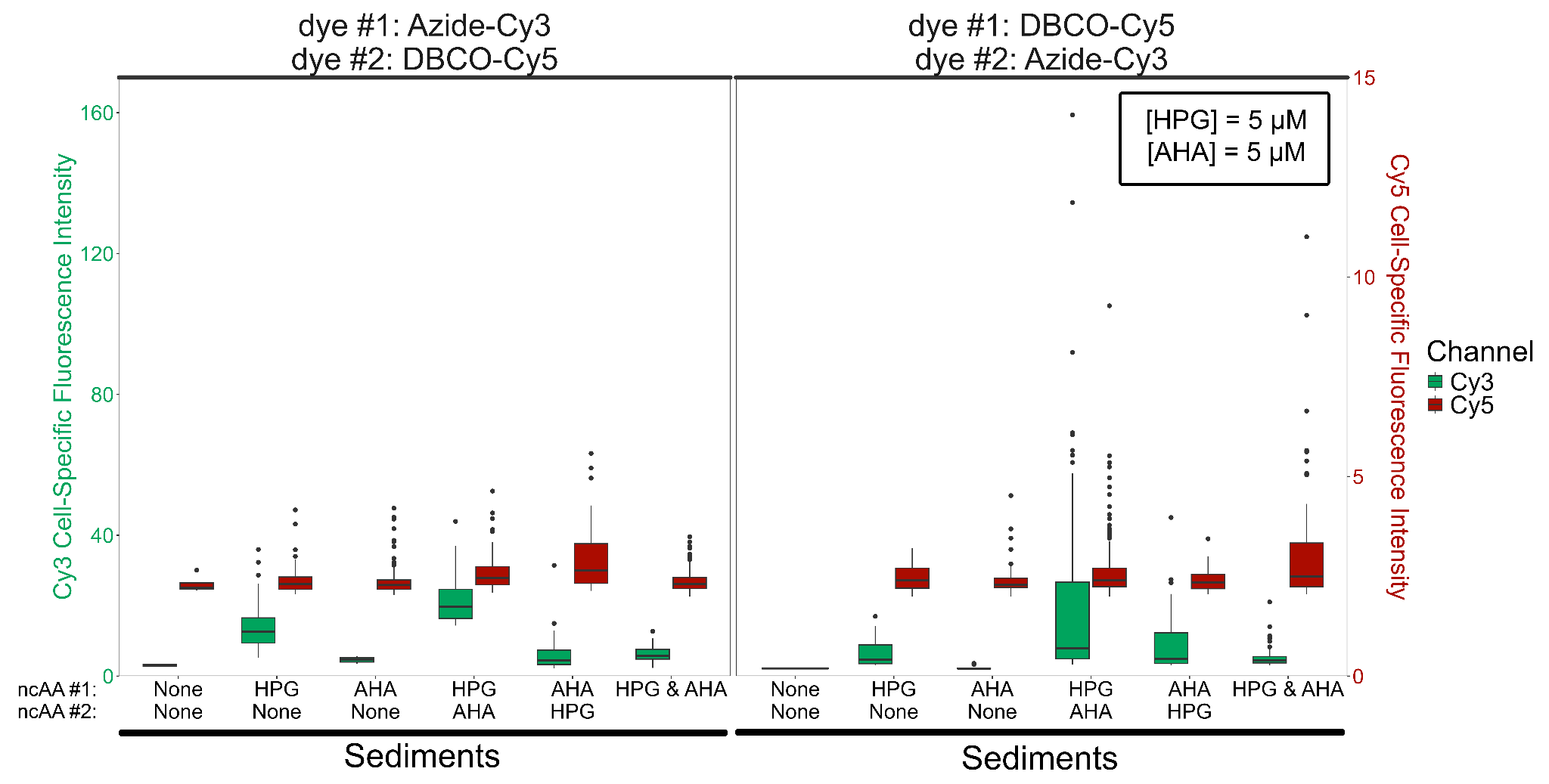


Fig. S11: Fluorescence microscopy field of view of a dual-BONCAT experiment with extracted salt marsh sediment cells. Both ncAAs (50 µM HPG & 500 µM AHA) were added at the start of the experiment, which was stopped after a single, 24-hour day-night cycle. The Azide-Cy3 click reaction was performed first. Scale bar indicates 5 µm.


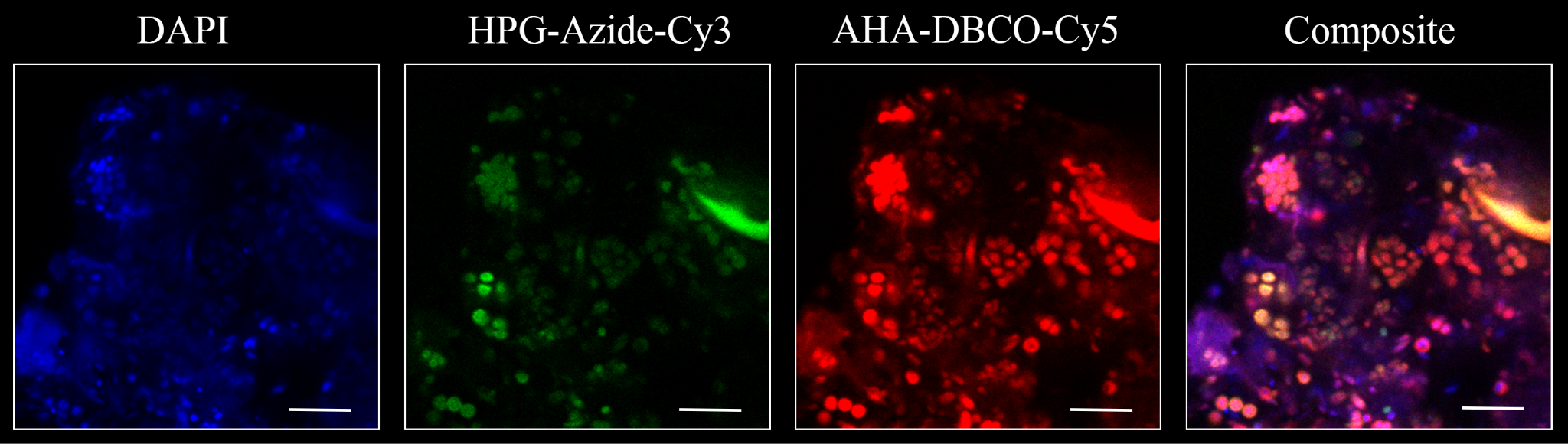


Fig. S12: An interpretive scheme for dual-BONCAT experiments. For a generalized experimental timeline during which one ncAA is added at an initial time point (T_1_) and a second ncAA is added at a subsequent time point (T_2_), the three distinct subpopulations of labeled cells (e.g., those labeled with only ncAA#1, both ncAA#1&2, and only ncAA#2) can be interpreted as being anabolically active during distinct phases of the experiment. Further details are provided in the text.


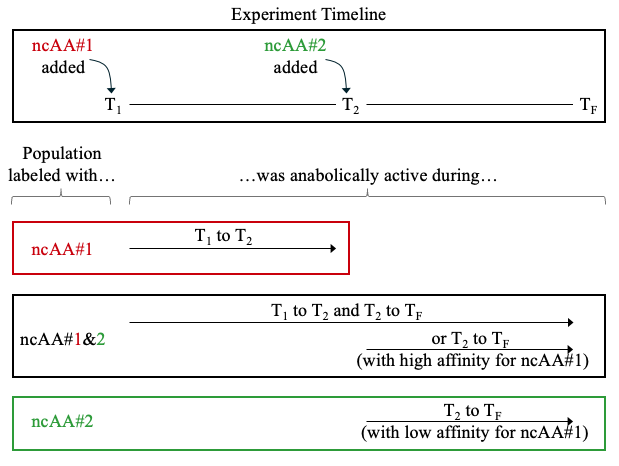


Fig. S13: Hypothetical ncAA concentrations during a dual-BONCAT experiment. Unless ncAAs are measured or removed, we cannot assume that ncAA#1 is entirely consumed by the time ncAA#2 is added at T_2_. Therefore, some dual-labeled microorganisms may have only been active from T_2_ to T_F_ while they still had access to ncAA#1.


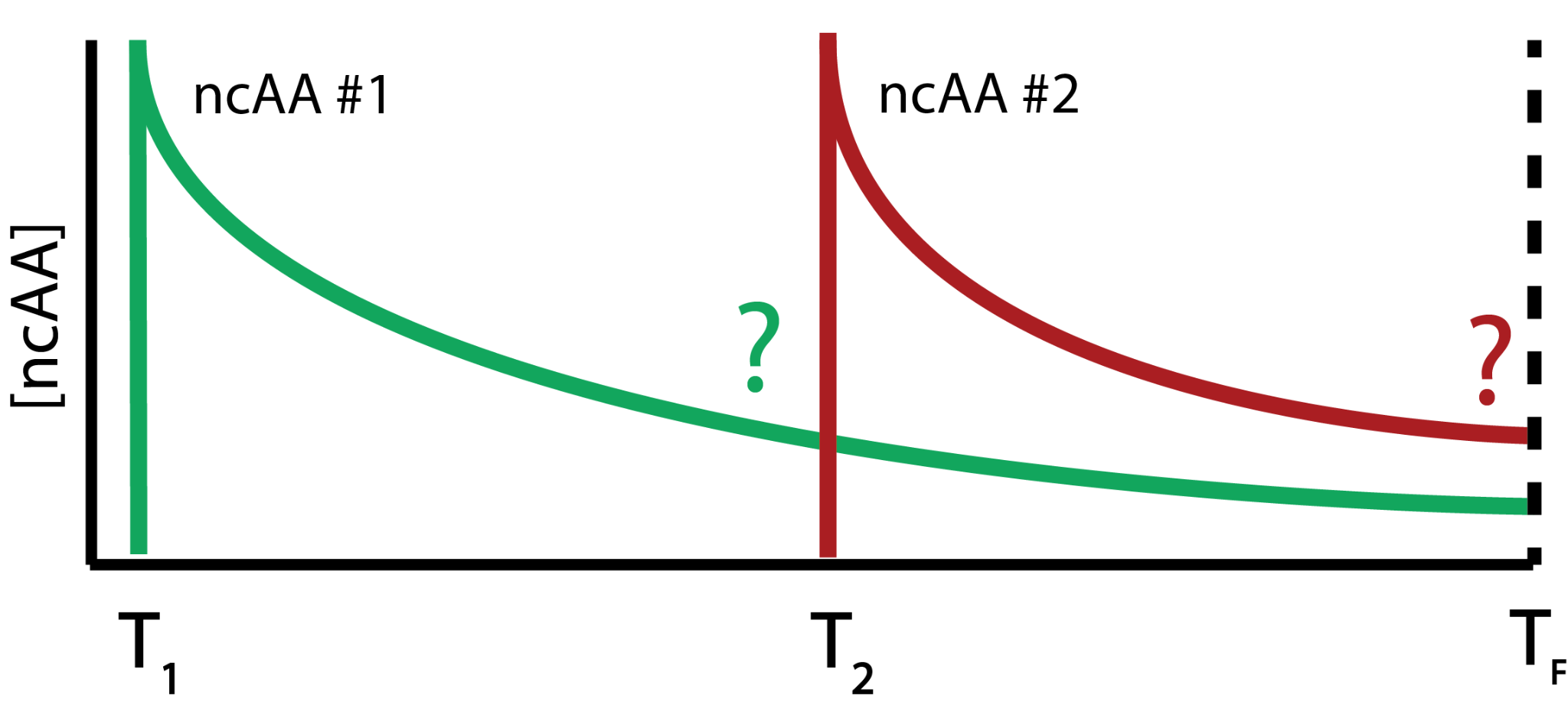


Fig. S14: Schematic representation of the subpopulations gating protocol. When two plots with different axes share the same numbering, it indicates that the same subpopulation should be identified. (1) Gate single cells subpopulation on a trigger pulse width versus forward scatter plot. The following gates are nested under gate 1, and this is the only nesting that was applied. (2) Gate Cy3 negative subpopulation by analyzing a ncAA-free sample with Cy3 and a Cy5 mono-labeled sample on the Cy3 plot [585/29 nm emission at 561 nm excitation versus forward scatter]. (3) Gate Cy3 positive subpopulation by analyzing a ncAA-Cy3 sample on the Cy3 plot and the Cy3Cy5 plot [692/40 nm emission at 640 nm excitation versus 585/29 nm emission at 561 nm excitation]. (4) Gate Cy5 negative subpopulation by analyzing a ncAA-free sample with Cy5 and a Cy3 mono-labeled sample on the Cy5 plot [692/40 nm emission at 640 nm excitation versus forward scatter]. (5) Gate Cy5 positive subpopulation by analyzing a ncAA-Cy5 sample on the Cy5 plot and the Cy3Cy5 plot. (6) Gate Cy3&Cy5 double-negative subpopulation by analyzing a ncAA-free sample with Cy3 and Cy5 on the Cy3Cy5 plot. (7) Gate Cy3&Cy5 double-positive subpopulation by analyzing a ncAA-Cy3&Cy5 sample on the Cy3Cy5 plot.


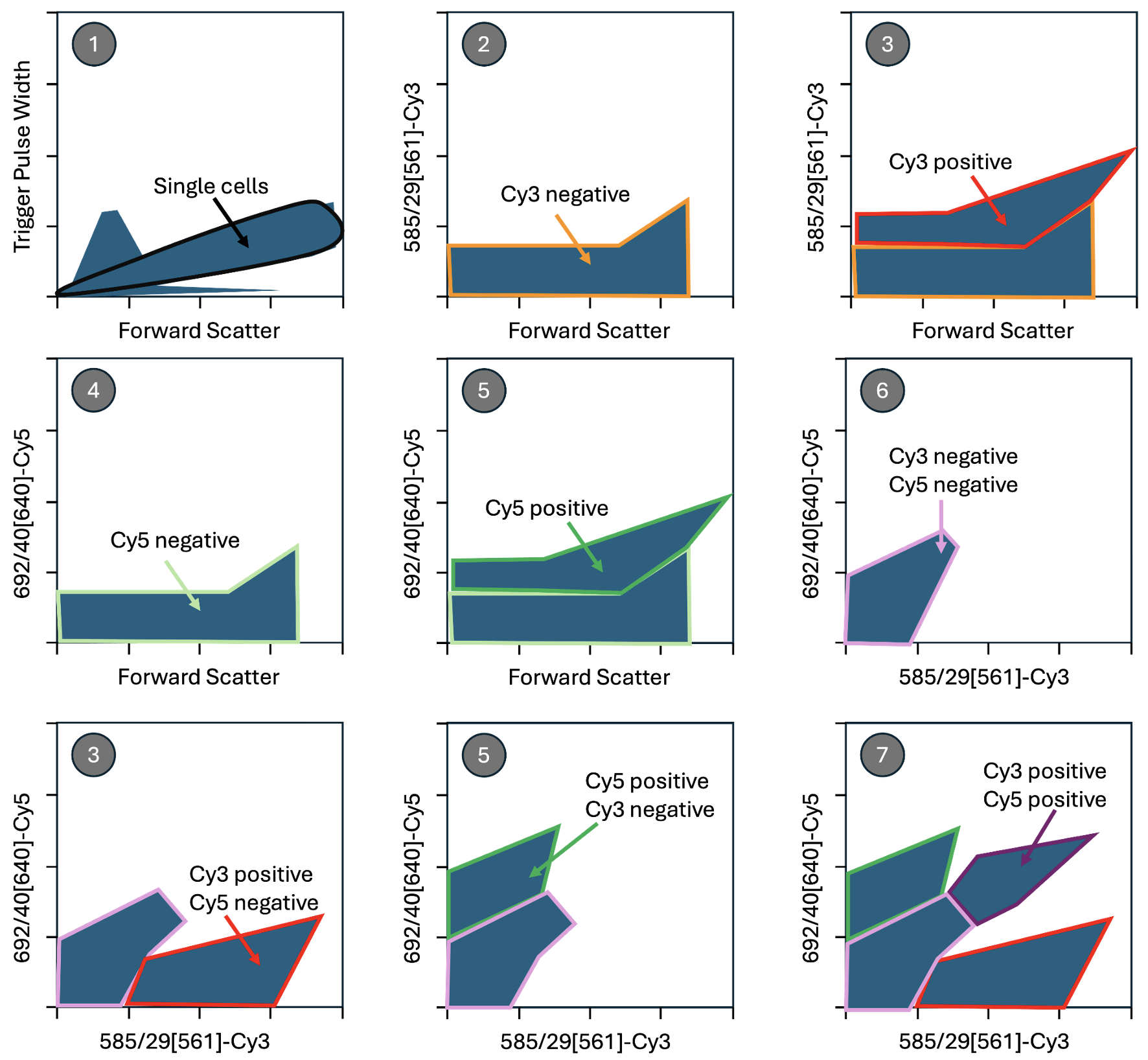


Table S1: Number of sorted cells recovered from each gate of interest in this study. Up to three cell sort replicates were pooled, as indicated, in order to recover additional biomass for downstream sequencing.


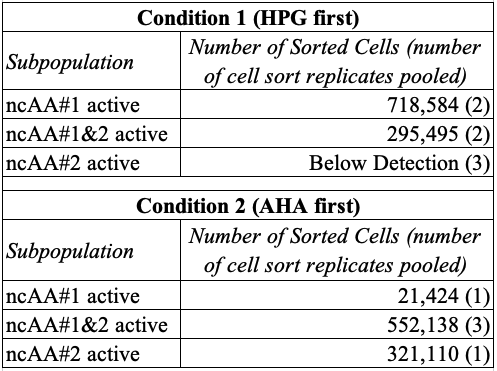


Table S2: Taxonomic identity and relative abundance of ASVs grouped at the family level for the seven communities sequenced in this study. Only family-level groupings that accounted for >1% of the relative abundance of at least one sequenced community are shown.

*See Excel File*

Table S3: Taxonomic identity and relative abundance of all ASVs for the seven communities sequenced in this study.

*See Excel File*

Table S4: Components of the two premixes needed for the Cu(I)-catalyzed HPG-azide reaction.


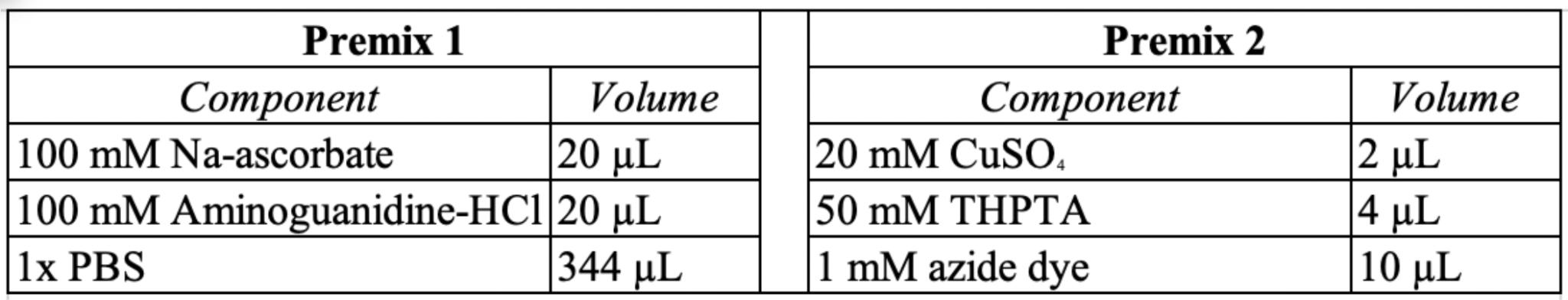


Table S5: Imaging parameters for the culture and environmental samples used in this study.


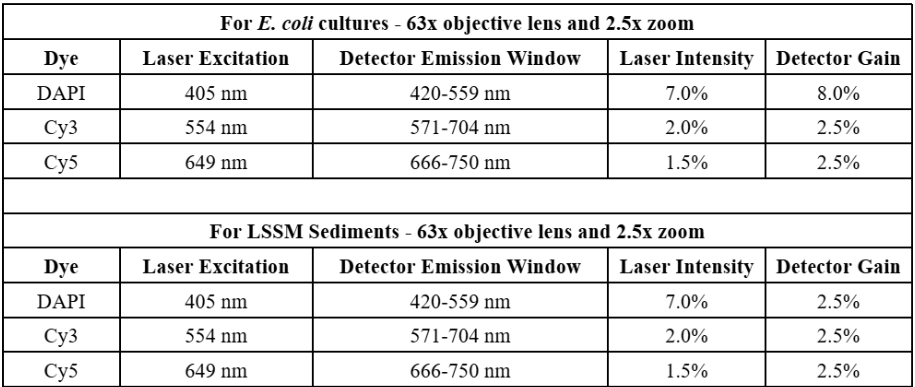


Table S6: FIJI Multi-Channel RGB Stacker plugin settings for the culture and environmental samples used in this study.


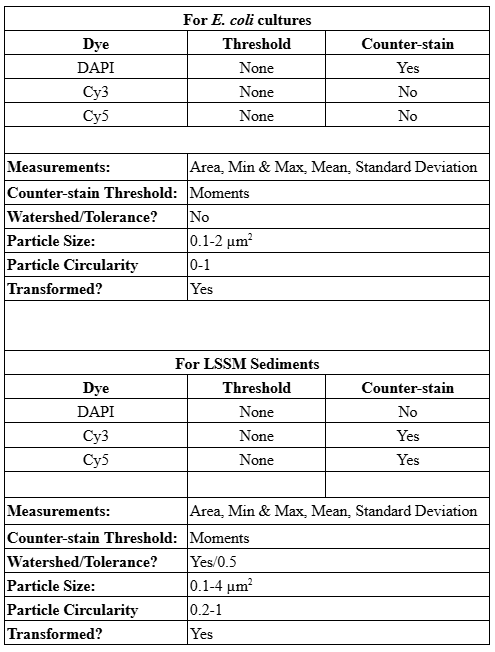


Table S7: FIJI Multi-Channel Cell Counter plugin settings for the culture and environmental samples used in this study.


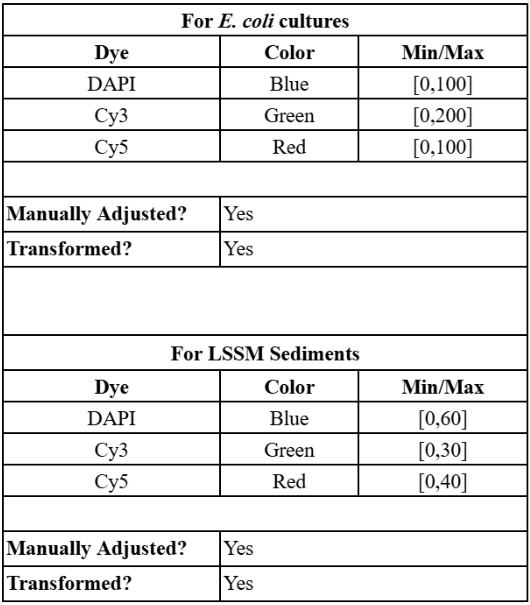


Table S8: Number of cells analyzed for the mono-BONCAT *E. coli* cultures. ROIs were chosen based on the DAPI counter-stain.


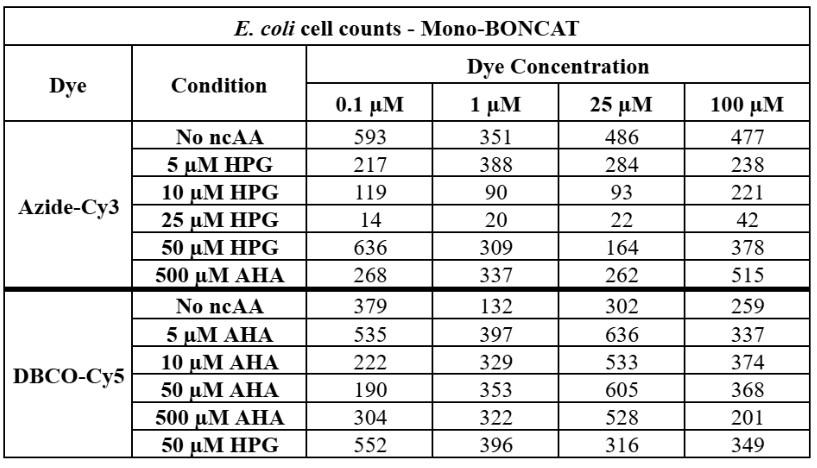


Table S9: Number of cells analyzed for the dual-BONCAT *E. coli* cultures. ROIs were chosen based on the DAPI counter-stain.


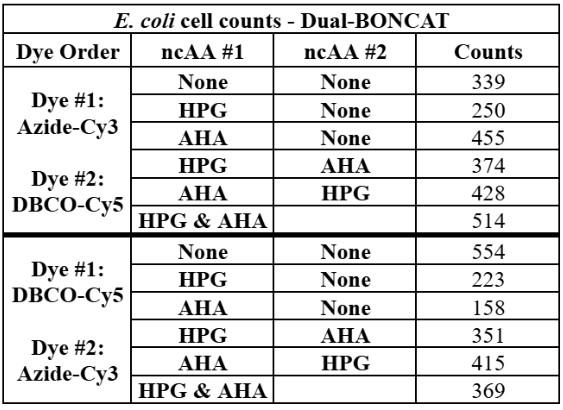


Table S10: Number of cells analyzed for the dual-BONCAT sediment experiments. ROIs were chosen based on the Cy3 and Cy5 channels, but only those that had an underlying mean DAPI intensity >5 were counted as cells.


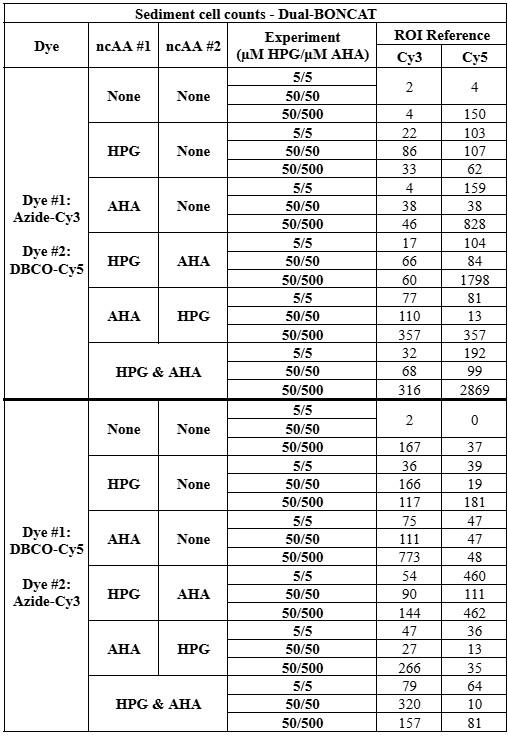


Table S11: Alpha diversity metrics for the seven sequenced communities analyzed in this study.


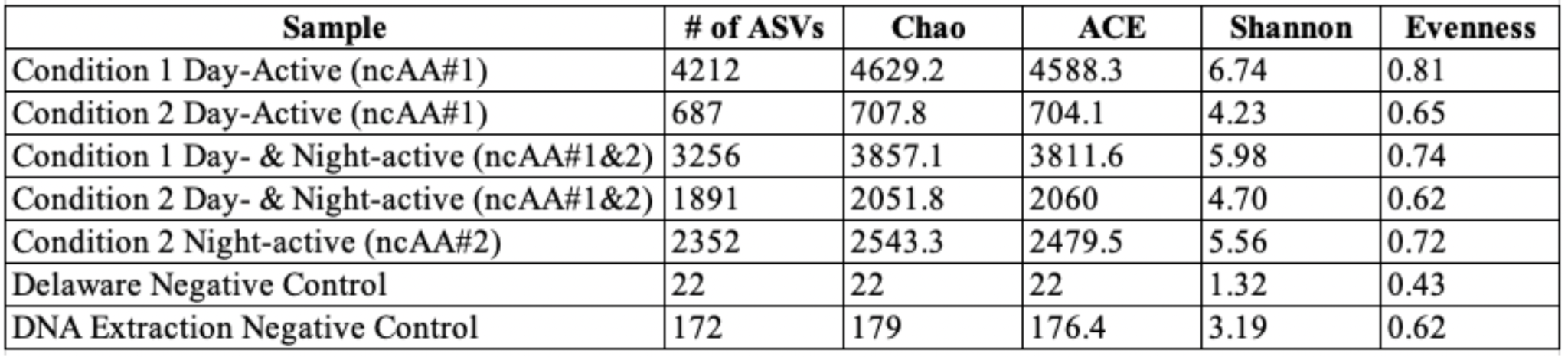

Supplement: Supplemental material — Fig. S1 to S14; Tables S1 and S4 to S11. [file aem.02391-25-s0001.docx]
